# Characterizing neonatal survival across the first four years of operation of a special care baby unit in Eastern Sierra Leone

**DOI:** 10.1186/s12887-026-07435-9

**Published:** 2026-08-04

**Authors:** Ashley Mitchell, Francess Kamara, Patricia Azikwe, Taofik Oluwaseun Ogunkunle, McGeofrey Mvula, Miranda Rouse, Kimberly Baltzell, Alden Hooper Blair

**Affiliations:** 1 Global Action in Nursing (GAIN), University of California, San Francisco, United States; 2 Institute for Global Health Sciences (IGHS), University of California, San Francisco, United States; 3 Koidu Government Hospital, Kono District, Koidu , Sierra Leone; 4 School of Nursing, University of California, San Francisco, United States

**Keywords:** Neonatal health, Special care baby units, Neonatal mortality, Sierra Leone

## Abstract

**Background:**

The recent establishment of Special Care Baby Units (SCBUs) across regional hospitals in rural Sierra Leone is an important advancement in health system infrastructure promoting high-quality perinatal care. However, there is little evidence documenting their impact. This study aimed to assess neonate-level characteristics and outcomes across the initial years of operation to inform clinical practices and policies.

**Methods:**

Neonatal chart data for infants with admission dates between June 8, 2021, and December 31, 2024, to the Kono District SCBU were collected by trained nurse researchers. Deidentified data were analyzed in RStudio. Descriptive summaries were conducted to map trends in diagnoses and bivariate analyses explored patterns by year of admission as well as by survival status.

**Results:**

A total of 3,857 neonatal patients were admitted during the study period with the most common primary diagnoses being ‘risk of neonatal sepsis or infection’ (*N* = 1,223, 32%), other (*N* = 606, 16%), and prematurity (*N* = 434, 11.25%). While there were no significant differences in birthplace by admission year, there were statistically significant fluctuations in referrals, delivery mode, birthweight, infant age at admission, and stay length. There was also a trend of increasing deaths over time with 16 (3%) neonatal deaths in 2021 and 56 (6%) deaths in 2024 (*p* = 0.042). Asphyxia, very low birthweight, and prematurity were the diagnoses with the highest likelihoods of death (up to 20%) while risk of neonatal sepsis or infection was the only diagnosis associated with survival (OR: 2.926, 95%CI: 1.945–4.592, *p* < 0.001).

**Conclusions:**

The early years of SCBU operation saw wide variation in neonatal characteristics and outcomes. Key shifts including the increase in referrals of younger patients may have contributed to the upward trend in deaths, though further exploration is needed. Overall, both the common diagnoses and key drivers of mortality aligned with national trends suggesting continued efforts to mitigate asphyxia and prematurity for SCBU patients are critical. As one of the first detailed characterizations of SCBU operation in a rural, low-income setting in this region, our findings underscore the importance of investing in timely, quality neonatal care.

## Background

The neonatal mortality rate (NMR) has steadily declined globally since 1990, driven by targeted medical advancements and public health initiatives, though regional disparities persist with higher rates in low-income countries including those in sub-Saharan Africa (SSA) [1–3]. While high-income countries including Australia and New Zealand report NMRs as low as 0.7 deaths per 1000 live births, the NMR in SSA is nearly 40 times this, at 27 deaths per 1000 live births [1, 4, 5]. With neonatal death accounting for nearly half of all under-5 mortality, defining and addressing the factors driving these disparities is critical [6]. Averting neonatal deaths is not only key to meeting international targets but NMR also plays a pivotal role in social and family structures as well as maternal health and wellbeing [5].

In Sierra Leone, the NMR has remained largely unchanged at 31 deaths per 1000 live births since 2021 [7, 8]. Many of the primary reported causes mirror leading causes globally and include prematurity and low birth weight (LBW), perinatal complications including birth asphyxia, and sepsis [9–11]. Indeed, national estimates suggest that about a third of neonatal deaths are caused by birth asphyxia, another 30% by sepsis and other infections, and just under one quarter are due to reasons related to prematurity [12]. Upstream, these causes are influenced by inadequate health system infrastructure which limits access to high-quality perinatal care. Comprehensive, patient-level data have rarely been analyzed from more rural regions—which account for the majority of the country—which continues to limit what is known.

There is a shortage of human and other resources limiting neonatal and maternal care in Sierra Leone. At an average of one doctor for every 10,000 people, the nation exceeds the World Health Organization’s (WHO) target by ten times, with even greater gaps for specialists such as neonatologists [13]. Additionally, it is estimated that more than half of the clinical staff in peripheral health facilities are unsalaried and essentially serve as volunteers, which leads to burnout, scheduling challenges, and provider shortages when staff members need to prioritize other income-generating activities [14, 15]. The effects of these provider-level issues are compounded by a widespread lack of essential equipment and medicines such as incubators and neonatal resuscitation supplies including oxygen [16, 17]. While access to and utilization of quality antenatal and perinatal services can help address downstream effects of care gaps, issues nonetheless remain. Women often travel long distances to reach health care facilities which can delay the receipt of care for their neonates—with disparities felt by rural residents most [18, 19].

Even when care is accessed and received, a variety of barriers continue to limit the provision of neonatal care. Prior research has noted delays in receiving care upon arrival to obstetric wards as well as delays and taboos in managing complications during pregnancy and birth [20–22]. There are extremely limited options for emergency neonatal care outside of major regional hospitals, and referral to these locations can be difficult due to seasonal transportation challenges of the rainy season [22, 23]. Finally, postnatal follow-up has been reported to be inconsistent with lower attendance among working mothers and those in more rural regions [8]. Improvements are needed across the neonatal care spectrum to reduce delays, strengthen care, and protect outcomes.

Across Sierra Leone, the neonatal health care landscape has evolved significantly over recent years toward overcoming persistent challenges. Some key interventions have included the construction and rehabilitation of eight emergency obstetric facilities, the establishment of Special Care Baby Units (SCBUs) in regional hospitals including the one in this study, and the training of 1,400 health care providers in essential newborn care [24–26]. These units are stocked with advanced medical equipment including infant warmers, phototherapy lamps, and emergency medications [25]. Still, recent research suggests that regional disparities persist [12, 18, 19]. Additionally, while accessibility of specialized neonatal care increases, there is a dearth of evidence documenting their successes and challenges [27, 28]. Improving understanding of the impact of these interventions will be key to systems strengthening and sustainability.

## Methods

This study contributes information on SBCU patient-level characteristics and outcomes in a rural and remote district of Sierra Leone. We summarized recent trends with the goal of informing clinical practices to strengthen neonatal outcomes. To characterize the initial years of operation for a newly established SCBU in Kono District in Eastern Sierra Leone, we analyzed neonatal charts for infants with admission dates between June 8, 2021, and December 31, 2024. The study was conducted by a binational team of nurses, nurse-midwives, and researchers at the University of California, San Francisco (UCSF) and Partners In Health (PIH), Sierra Leone who together make up a collaboration called Global Action in Nursing (GAIN).

### Study setting

The team collaborated with the Ministry of Health (MoH) in Sierra Leone since 2022 to elevate high-quality nursing and midwifery care. Our work has included short-course trainings and longitudinal bedside mentorship by expert Sierra Leonean nurse-midwives to improve maternal and neonatal outcomes. The MoH selected four peripheral health units (PHUs) and one tertiary hospital across Kono District for the collaboration, based on their higher volume of maternal and neonatal patients.

The first SCBU in Kono opened in August 2020 with support from UNICEF and the Government of Sierra Leone. Prior to opening, three providers in Kono received year-long neonatal health training in Makeni and Freetown through Njala University. Facility nurses were also trained in chart extraction to improve data integrity. The Kono SCBU has 20 neonatal beds, a dedicated Kangaroo Mother Care (KMC) ward, and a Mother’s Waiting Room. Notably, Continuous Positive Airway Pressure (CPAP) machines were not immediately available though were stocked over time. SCBU providers received training and mentorship to scale their expertise in critical neonatal and pediatric care [25]. Like many health care facilities, providers unfortunately describe staffing to be often inadequate compared to patient needs. On average, more than 100 neonatal patients are estimated to be seen each month. This is the first analysis of patient data since Kono SCBU’s opening.

### Data collection

Data were collected by trained facility nurses with routine access to patient charts. Key variables were extracted from all hard copy neonatal patient registers between the SCBU opening and the end of 2024 into a secure Excel spreadsheet. Entries were then reviewed by the local monitoring and evaluation (M&E) team to confirm quality and completeness. Deidentified data were shared with designated members of the research team for analysis. A full list of all diagnoses reported is listed in Appendix 1. These were reviewed with the SCBU clinical leads (authors: TOO, MM). Highly related variables were combined into one level on their suggestions based on clinical practice at the site.

### Data analysis

The primary goal was to gain a baseline understanding of patient characteristics, outcomes, and clinical metrics including diagnoses and stay length in the early years of SCBU operation. Data were extracted to RStudio for analyses [29]. Descriptive analyses were conducted to summarize diagnoses overall and by year as well as to map the relationships among patients with multiple diagnoses. Missing data were kept and reported as this varied by variable. Bivariate Fisher’s Exact tests were conducted to assess trends in neonate characteristics by year of SCBU admission. This included assessing for seasonal differences between the “wet” (July through October) and “dry” seasons of each year. Sub-analyses were then conducted among the neonates with a recorded outcome to assess for differences based on survival status. For this, we combined outcomes of “Discharged”, “Absconded”, and “Referred Out” into a single category considered to have survived their admitted time in the SCBU and compared it to those with a known death—these outcome categories reflect local discharge practices and reporting. For all analyses, we considered a *p*-value of < 0.05, or odds ratio with a 95% confidence interval that did not cross 1 to be significant.

### Ethical considerations

The study was approved by the Sierra Leone Ethics and Scientific Review Committee (SLERC) (035/01/2025), and UCSF Human Research Protection Program (HRPP) (23-38364).

## Results

A total of 18 different diagnosis categories, including ‘other’, were recorded across the first four years of SCBU operation (Table 1). The majority (*N* = 3,842, > 99%) of neonates had at least one primary diagnosis in their chart, with 2,603 (67%) having at least two diagnoses, and 1,081 (28%) with three reported diagnoses. The most common primary diagnosis was ‘risk of neonatal sepsis or infection’ (*N* = 1,223, 32%) followed by ‘other’ (*N* = 606, 16%), which includes congenital abnormalities and birth injuries among other less common diagnoses, and prematurity (*N* = 434, 11.25%). The most common secondary diagnosis was also risk of neonatal sepsis or infection (*N* = 583, 15%) followed by ‘other’ (*N* = 487, 19%), and low birthweight (*N* = 424, 11%). Only the other category of diagnoses impacted more than 10% of neonates as the third diagnosis.

**Table 1:** Summary of neonate diagnoses across SCBU admissions

Diagnosis	Primary Dx		Secondary Dx		Third Dx	
	#	%	#	%	#	%
Birth Asphyxia	280	7.44%	104	2.70%	41	1.06%
Low Birthweight	230	5.96%	424	10.99%	90	2.33%
Very Low Birthweight	53	1.37%	74	1.92%	14	< 1.00%
Extremely Low Birthweight	4	< 1.00%	6	< 1.00%	4	< 1.00%
HIV Exposure	64	1.66%	32	< 1.00%	22	< 1.00%
Risk of Neonatal Sepsis or Infection	1,223	31.78%	583	15.12%	362	9.39%
Neonatal Sepsis	539	14.03%	234	8.99%	93	8.61%
Malaria	71	1.84%	64	1.66%	18	< 1.00%
Neonatal Jaundice	90	2.33%	120	3.11%	46	1.19%
Meningitis	9	< 1.00%	7	< 1.00%	0	0.00%
Macrosomia	29	< 1.00%	1	< 1.00%	0	0.00%
Respiratory Distress Syndrome	250	6.48%	337	8.74%	166	4.30%
Prematurity	434	11.25%	160	4.15%	51	1.32%
Other ^a^	606	15.77%	487	18.71%	195	18.04%
No Diagnosis or NA ^b^	15	< 1.00%	1,254	32.51%	2,776	71.97%

^a^“Other” is a dx recorded by SCBU providers in patient charts and includes congenital abnormalities, birth injuries, acute respiratory infection, fever, and other less common diagnoses

^b^No Diagnosis for Primary Diagnosis and NA for Secondary and Tertiary

The use of each diagnosis except four varied significantly by admission year (Table 2, Appendix 2). Low birthweight, very low birthweight, extremely low birthweight, and neonatal jaundice were the only diagnoses with similar percentages each year. While some of the significant changes in diagnoses, such as birth asphyxia—which accounted for 16% of diagnoses in 2021, dropping below 10% in 2022 and 2023, and increasing again in 2024, went up and down across years (*p* < 0.001)—others had a more prominent directional trend. ‘Risk of neonatal sepsis or infection’, for instance, made up more than 80% of diagnoses in 2021 which steadily reduced to less than 30% by 2024 (*p* < 0.001). The first year had no recorded diagnoses of neonatal sepsis, neonatal jaundice, meningitis, or macrosomia—the final of which was the only diagnosis to occur in just one admission year (2024) (*p* < 0.001). Altogether, there were seven or fewer neonates without any recorded diagnosis each year.

**Table 2:** Summary of diagnoses a by admission year

Admission Year	2021^b^ (*N* = 568) *n* (%)	2022 (*N* = 1,335) *n* (%)	2023 (*N* = 1,052) *n* (%)	2024 (*N* = 902) *n* (%)	*p*-value*
Birth Asphyxia	90 (15.8%)	132 (9.9%)	95 (9.0%)	106 (11.8%)	**< 0.001**
Low (1500–2499 g)	99 (17.4%)	265 (19.9%)	202 (19.2%)	177 (19.6%)	0.662
Very Low (1000- 1499 g)	13 (2.3%)	43 (3.2%)	50 (4.8%)	35 (3.9%)	0.059
Extremely Low (< 1000 g)	4 (0.7%)	2 (0.1%)	4 (0.4%)	4 (0.4%)	0.243
HIV Exposure	5 (0.9%)	50 (3.7%)	39 (3.7%)	24 (2.7%)	**< 0.001**
Risk of Neonatal Sepsis or Infection	469 (82.57%)	887 (66.44%)	418 (39.73%)	262 (29.05%)	**< 0.001**
Neonatal Sepsis	0 (0.0%)	271 (20.30%)	392 (37.26%)	218 (24.17%)	**< 0.001**
Malaria	19 (3.3%)	67 (5.0%)	38 (3.6%)	29 (3.2%)	0.122
Neonatal Jaundice	0 (0.0%)	62 (4.6%)	126 (12.0%)	67 (7.4%)	**< 0.001**
Meningitis	0 (0.0%)	3 (0.2%)	0 (0.0%)	13 (1.4%)	**< 0.001**
Macrosomia	0 (0.0%)	0 (0.0%)	0 (0.0%)	30 (3.3%)	**< 0.001**
Respiratory Distress Syndrome	144 (25.4%)	291 (21.8%)	197 (18.7%)	121 (13.4%)	**< 0.001**
Prematurity	68 (12.0%)	240 (18.0%)	185 (17.6%)	151 (16.7%)	**0.008**
Other ^c^	185 (32.6%)	428 (32.1%)	299 (28.4%)	313 (34.7%)	**0.026**
No Diagnosis	0 (0.0%)	3 (0.2%)	5 (0.5%)	7 (0.8%)	0.080

*p-value bolded if < 0.05

^a^Combines first, second, and third diagnoses categories to be inclusive of ever-diagnosed; note that some infants were diagnosed with varying levels of a collapsed diagnosis (i.e. Birth Asphyxia I and later Birth Asphyxia II) and those cases will only be counted once here

^b^Note that 2021 only includes the latter 6-months given the timing of SCBU opening

^c^Other includes congenital abnormalities, birth injuries, acute respiratory infection, hyperthermia, and other less common diagnoses

Further exploration of the relationship between primary diagnoses and additional diagnoses by neonate, offered some additional insight (Appendix 3). For example, among those with a primary diagnosis of Sepsis Risk, 508 (44%) had no secondary diagnosis. For those with a primary diagnosis of Prematurity or Birth Asphyxia on the other hand, 341 (97%) and 133 (82%) respectively had at least one additional diagnosis. A similar trend is seen for neonates with primary diagnoses of Low Birthweight and Asphyxia.

Between the opening of the SCBU in 2021 and December 2024, 3,857 neonatal patients were admitted (Table 3). To date, the highest number of neonatal patients (*n* = 1,335) was in 2022. Among the charts reviewed, there were notable trends by year of SCBU admission. While there were no significant differences in birthplace, the highest proportion of referrals from home (*N* = 180, 32%) occurred in 2021, and the highest proportion of internal hospital referrals (*N* = 702, 67%) occurred in 2023 (*p* < 0.001). Delivery mode also fluctuated significantly (*p* = 0.008) by year, as the percentage of neonates born via cesarean who ended up in the SCBU gradually decreased from 47% to 42% over the first four years and uncomplicated vaginal births saw a corresponding overall increase from 51% to 58%. Additionally, while the proportion of neonates with extremely low (< 1000 g) birthweight remained stable, the proportion of low and very low birthweight infants has increased over time (*p* < 0.001). Infant age (in days) at admission also decreased over time from an average of five days (SD: 8.05) in 2021 to about 2.5 days (SD: 5.30) in 2024 (*p* < 0.001) with the longest average stay length occurring in 2022 (8.15 days, SD: 6.28, *p* < 0.001). Finally, there was an increasing proportion of infant deaths each year—while there were 16 (3%) neonatal deaths in 2021, this gradually increased to 56 (6%) deaths in 2024 (*p* = 0.042).

**Table 3:** Neonate characteristics by year of SCBU admission

	2021 *n* = 568 (14.73%)	2022 *n* = 1,335 (24.61%)	2023 *n* = 1,052 (27.28%)	2024 *n* = 902 (23.39%)	*p*-value*	Overall *N* = 3,857
Birthplace					0.300	
Hospital	424 (74.65%)	1,029 (77.08%)	786 (74.71%)	659 (73.06%)		2,898 (75.14%)
Peripheral Health Unit (PHU)	116 (20.42%)	229 (17.15%)	204 (19.39%)	198 (21.95%)		747 (19.37%)
Home	26 (4.58%)	74 (5.54%)	59 (5.61%)	43 (4.77%)		202 (5.24%)
Other Districts	1 (< 1%)	3 (< 1%)	3 (< 1%)	1 (< 1%)		8 (< 1%)
Missing	1 (< 1%)	0 (0%)	0 (0%)	1 (< 1%)		2 (< 1%)
Referred From					**< 0.001**	
Internal (Hospital Maternity)	345 (60.74%)	866 (64.87%)	702 (66.73%)	592 (65.63%)		2,505 (64.95%)
PHU	42 (7.39%)	109 (8.16%)	112 (10.65%)	59 (6.54%)		322 (8.35%)
Home	180 (31.69%)	360 (26.97%)	232 (22.05%)	249 (27.61%)		1,021 (26.47%)
Other Districts	0 (0%)	0 (0%)	4 (< 1%)	1 (< 1%)		5 (< 1%)
Missing	1 (< 1%)	0 (0%)	2 (< 1%)	1 (< 1%)		4 (< 1%)
Delivery Mode					**0.008**	
Uncomplicated Vaginal	287 (50.53%)	638 (47.79%)	596 (56.65%)	520 (57.65%)		2,041 (52.92%)
Cesarean	271 (47.71%)	604 (45.24%)	452 (42.97%)	380 (42.13%)		1,707 (44.26%)
Vacuum	0 (0%)	0 (0%)	1 (< 1%)	1 (< 1%)		2 (< 1%)
Missing	10 (1.76%)	93 (6.97%)	3 (< 1%)	1 (< 1%)		107 (2.77%)
Birthweight					**< 0.001**	
Normal (> 2499 g)	380 (66.90%)	856 (64.12%)	633 (60.17%)	507 (56.21%)		2,376 (61.60%)
Low (1500–2499 g)	157 (27.64%)	395 (29.59%)	336 (31.94%)	318 (35.25%)		1,206 (31.27%)
Very Low (1000- 1499 g)	18 (3.7%)	69 (5.17%)	63 (5.99%)	67 (7.43%)		217 (5.63%)
Extremely Low (< 1000 g)	7 (1.23%)	12 (< 1%)	17 (1.62%)	7 (< 1%)		43 (1.11%)
Missing	6 (1.06%)	3 (< 1%)	3 (< 1%)	3 (< 1%)		15 (< 1%)
Infant Age in Days at Admission					**< 0.001**	
Mean (SD)	5.03 (8.05)	4.85 (8.03)	3.73 (7.18)	2.45 (5.30)		4.01 (7.31)
Missing	0 (0%)	1 (< 1%)	1 (< 1%)	0 (0%)		2 (< 1%)
Infant Stay Length					**< 0.001**	
Mean (SD)	6.80 (5.38)	8.15 (6.28)	5.66 (5.21)	5.78 (4.42)		6.74 (5.58)
Missing	80 (14.08%)	91 (6.82%)	126 (11.98%)	67 (7.43%)		
Outcome					**0.042**	
Discharged	476 (83.80%)	1,164 (87.19%)	877 (83.37%)	775 (85.92%)		3,292 (85.35%)
Referred Out	1 (< 1%)	1 (< 1%)	2 (< 1%)	1 (< 1%)		5 (< 1%)
Absconded	0 (0%)	0 (0%)	1 (< 1%)	0 (0%)		1 (< 1%)
Died	16 (2.82%)	57 (4.27%)	58 (5.51%)	56 (6.21%)		187 (4.85%)
Missing	75 (13.20%)	113 (8.46%)	114 (10.84%)	70 (7.76%)		372 (9.64%)

*p-value bolded if < 0.05

Among the subset of data for neonates who had a known outcome (*N* = 3,485, 90%), there were no significant differences in survival by infant sex (*p* = 0.446), maternal age (*p* = 0.187), birthplace (*p* = 0.637), delivery mode (*p* = 0.220), Hb (*p* = 0.532), or by rainy/dry season (*p* = 0.138). However, infant age was higher (mean: 4.25 days, SD: 7.51) among those who survived compared to those who died (mean: 1.38, SD: 3.91) (*p* < 0.001). Similarly, infants who survived had a longer stay on average (mean: 6.74, SD: 5.41) compared to those who died (mean: 5.30, SD: 6.66) (*p* < 0.001). We also found a corresponding increase in death over time by admission year (*p* = 0.018) (Table 4). Neonates who were referred from home were the most likely to survive (97%) compared to those referred from PHUs (93%) or internally from the hospital (94%) (*p* = 0.011). Neonates had a decreasing chance of survival by birthweight with 97% of those at “Normal” (> 2499 g) birthweight surviving and only 52% of those at an Extremely Low (< 1000 g) birthweight surviving (p-value included for consistency).

**Table 4:** Admission characteristics by survival status

	Survival *N* = 3,298 (94.63%)	Death *N* = 187 (5.37%)	OR	95% CI	*p*-value
Admission Year					0.018
2021	477 (96.75%)	16 (3.25%)	*ref*	*ref*	
2022	1,165 (95.34%)	57 (4.66%)	0.686	0.378, 1.177	
2023	880 (93.82%)	58 (6.18%)	0.509	0.280, 0.874	
2024	776 (93.27%)	56 (6.73%)	0.465	0.255, 0.800	
Referred From					**0.011**
Home	911 (96.61%)	32 (3.39%)	*ref*	*ref*	
Internal ^a^	2,123 (93.94%)	137 (6.06%)	0.544	0.362, 0.795	
PHU	256 (93.43%)	18 (6.57%)	0.500	0.279, 0.922	
Other Districts	4 (100.00%)	0 (0.00%)	*NA*	*NA*	
Missing	4 (100.00%)	0 (0.00%)	*NA*	*NA*	
Birthweight					**< 0.001**
Normal (> 2499 g)	2,147 (96.62%)	75 (3.38%)	*ref*	*ref*	
Low (1500–2499 g)	1,001 (93.46%)	70 (6.54%)	0.500	0.358, 0.699	
Very Low (1000–1499 g)	125 (81.70%)	28 (18.30%)	0.156	0.098, 0.253	
Extremely Low (< 1000 g)	14 (51.85%)	13 (48.15%)	0.038	0.017, 0.084	
Missing	11 (94.63%)	1 (5.37%)	*NA*	*NA*	

^a^Admitted directly from Hospital Maternity Ward

*p - value bolded if < 0.05

Finally, we looked at relationships between primary diagnoses and corresponding neonatal outcome and found a variety of notable differences (Appendix 4). Neonates with a diagnosis of birth asphyxia had a 20% likelihood of death compared to a 4% likelihood among those without this diagnosis (OR: 0.211, 95%CI: 0.147–0.307, *p* < 0.001). Additionally, 17% of those with a primary diagnosis of very low birthweight (1000–1499 g) died, compared to 5% of those without this primary diagnosis (OR: 0.268, 95%CI: 0.124–0.666, *p* = 0.005). Furthermore, infants diagnosed with prematurity were 5% less likely to survive compared to those without this diagnosis (OR: 0.463, 95%CI: 0.319–0.691, *p* < 0.001). Interestingly, the only diagnosis that was associated with a higher likelihood of survival was risk of neonatal sepsis or infection as 98% of neonates with this as their primary diagnosis survived compared to 93% who had other primary diagnoses (OR: 2.926, 95%CI: 1.945–4.592, *p* < 0.001).

## Discussion

The early years of SCBU operation in the Kono District of Sierra Leone saw a wide range of diagnoses and clinical metrics. Risk of neonatal sepsis, neonatal sepsis itself, and prematurity were among the most common diagnoses, aligning with national and global statistics [9, 10, 12]. However, about a third of diagnoses each year were recorded as “other.” Current providers suggested that these were likely to include congenital abnormalities, birth injuries, acute respiratory infection, fever, and other less common diagnoses. Standardized registers were only implemented in early 2024, which are likely to ensure more standardized recording of diagnoses as well as less missingness overall. Regardless of these challenges, the results provide critical insight into the clinical needs of neonates in rural and remote settings of sub-Saharan Africa.

The growth of the SCBU, as well as knowledge of its availability and services, likely provided both benefits and challenges to neonatal care in the region. As local facilities, as well as those further afield, became more familiar, internal referrals and facility transfers increased. While this reduced the average admission age as facilities directly referred patients earlier, it also likely increased the caseload and acuity of patients. This may help explain the upward trend in infant deaths, where neonates who may otherwise have died at peripheral facilities, home, or following care in the hospital were instead sent to the SCBU. Future analyses should explore trends of neonatal mortality in these other settings to see if corresponding decreases are seen.

It is worth noting that the 5% death rate observed in our study, was only slightly higher than the national NMR of 30.8 deaths per 1,000 live births, recorded in 2022—which increased from 29.6 deaths per 1,000 live births in 2019, despite a decade of reductions prior to this [18, 30]. Additionally, the national rate accounts for all births in the country, not those more at risk due to their identified need for specialized neonatal care as the case in our study. The rate is lower than neonatal intensive care facilities in similar settings, several of which report mortality rates between 15–20% [31, 32].

It is also notable that the opening of the SCBU overlapped with health care disruptions due to the global COVID-19 pandemic. While the full impact of this cannot be measured, we do know that this likely impacted metrics including HIV testing in 2021 [33]. The increase in neonatal HIV exposure identified between 2021 and 2022 in our study may not, therefore, be a true increase and may instead reflect a restoration of routine testing. Finally, between August and November of 2023 and again in early 2024, all infants in the SCBU were evacuated to three maternity unit rooms due to infection outbreaks. Deep cleaning was then done to mitigate infection, and while necessary, this likely resulted in more crowding and reduced timely access to equipment which may have impacted how care could be provided and, inevitably, the outcomes as seen in the data.

The causes of mortality among SCBU patients align with national trends and were associated with asphyxia (*N* = 44) or prematurity (*N* = 35) [12]. Prematurity trends remain stubborn at least in part due to the prominence of maternal hypertensive disorders nationally, anemia during pregnancy, and a less than 2 years inter-pregnancy interval, as well as persistent risks of malaria in pregnancy [34, 35]. Continued upstream efforts to ensure full-term births remain critical, and health care providers are being encouraged to screen and sensitize mothers on the risk factors of prematurity during antenatal sessions.

The increased survival for patients who remained in the SCBU for longer periods of time makes sense logically as the most ill or vulnerable experience higher risk of death (“depletion of susceptibles”). It also aligns with other research in the region which found the highest risk of death to be within the first four days of life [36]. Similarly, the increased survival for those referred from home could correspond to a higher perceived baseline healthiness, as they were initially discharged from the hospital or health center rather than immediately referred to the SCBU. Because of this sequence, those referred from home were also slightly older upon admission on average, likely influencing their chance at survival even if they were among the 5% born at home.

Among diagnoses, the only one associated with a greater likelihood of survival was ‘risk of neonatal sepsis.’ While broadly defined, providers at the SCBU suggested this was due to reported infection outbreaks during the study period. This diagnosis historically served as a catch-all for infants who were relatively stable upon admission but experienced maternal and perinatal risk factors for neonatal infections including premature rupture of membranes, prolonged labor, delivery through meconium-stained amniotic fluid, resuscitation at birth, low APGAR scores, and/or maternal fever. Infants with this diagnosis receive routine antibiotics for several days in addition to generally increased monitoring which may be protective overall. Over time this diagnosis was used less frequently as SCBU providers grew in their confidence to recognize specific conditions and improve clinical diagnostic certainty.

Our study contributes one of the first detailed characterizations of the early years of operation of an SCBU in a rural, low-income setting. Our binational team of clinicians and researchers strengthened our ability to summarize and interpret these findings. We faced several limitations, including the lack of quality documentation and missingness across variables including infant outcomes. In general, there is a lot of room for improvement when it comes to SCBU data collection and quality. Future research utilizing the recently implemented registers will be critical to filling these gaps. Additionally, leadership and clinician changes across the four years of data collection may also have changed the way metrics were recorded or given in ways that were not able to be fully captured here.

Clinically, the SCBU would benefit from focused interventions to manage asphyxia and prematurity as drivers of risk and poor outcomes. At the same time, efforts to improve upstream prevention of these conditions through bolstered antenatal care and prompt resuscitation across health facilities need to be reinforced. As referrals remain steady or increase, training to support effective triage and treatment on both ends of care transfers also remains key. Finally, continued professional development opportunities to improve data collection and quality improvement would ensure gains are measurable and sustainable [37]. Further longitudinal studies would inform resource allocation at rural neonatal care settings, like the one in our study.

## Conclusion

These findings indicate the continued importance of investing in timely, quality neonatal care across low-income settings, particularly for infants with conditions associated with a risk of death including asphyxia and prematurity. Challenges persist in settings without full-capacity Neonatal Intensive Care Units that results in what would be otherwise preventable deaths. Particularly as healthcare utilization increases, upstream efforts by the MoH and similar stakeholders to ensure adequate, skilled staffing and space will be key. Additional attention should be paid to trends in neonatal survival to better understand risk and protective factors—sustained and improved quality data collection is necessary for this. Ensuring high quality neonatal care and averting deaths not only makes progress toward international targets but strengthens social and family structures to bolster health equity.

## Figures and Tables

**Fig. 1:**
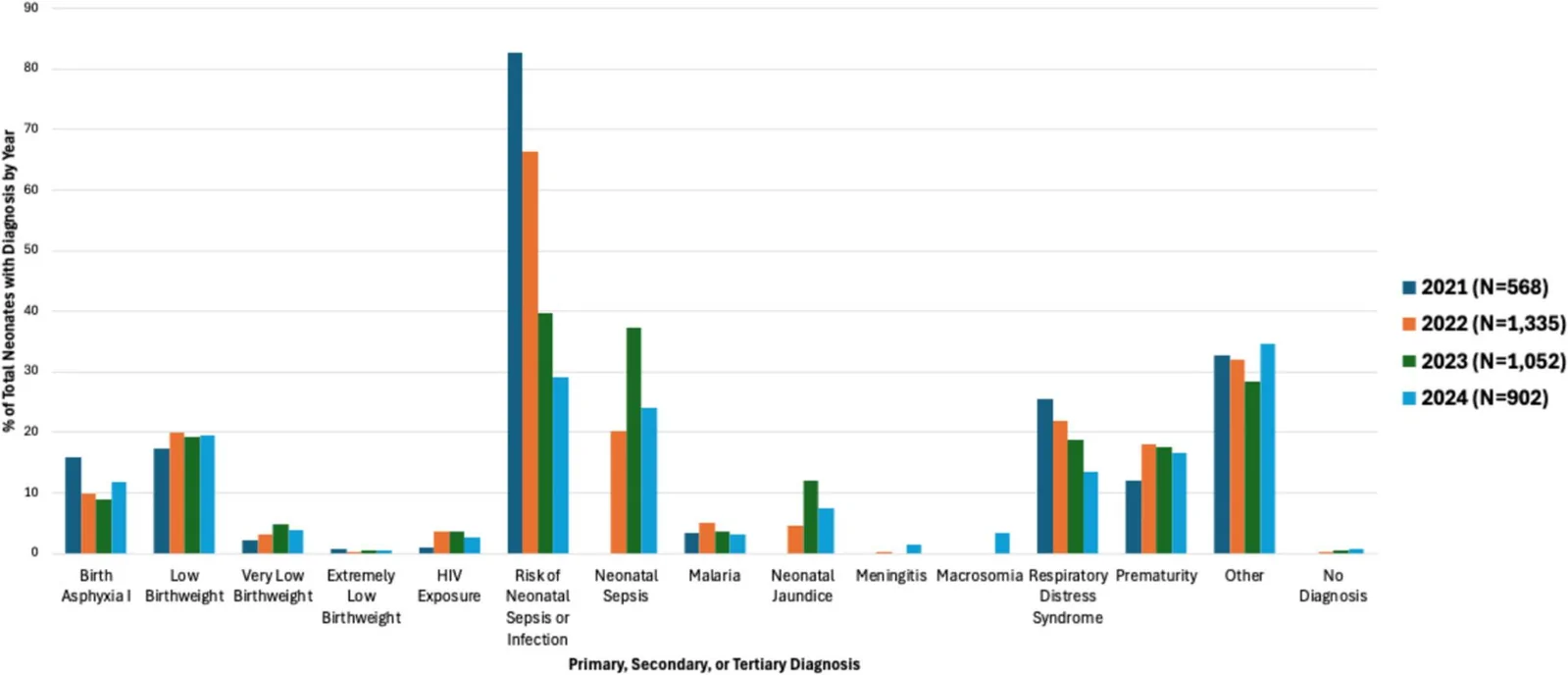
Neonatal diagnoses by year of admission

**Fig. 2:**
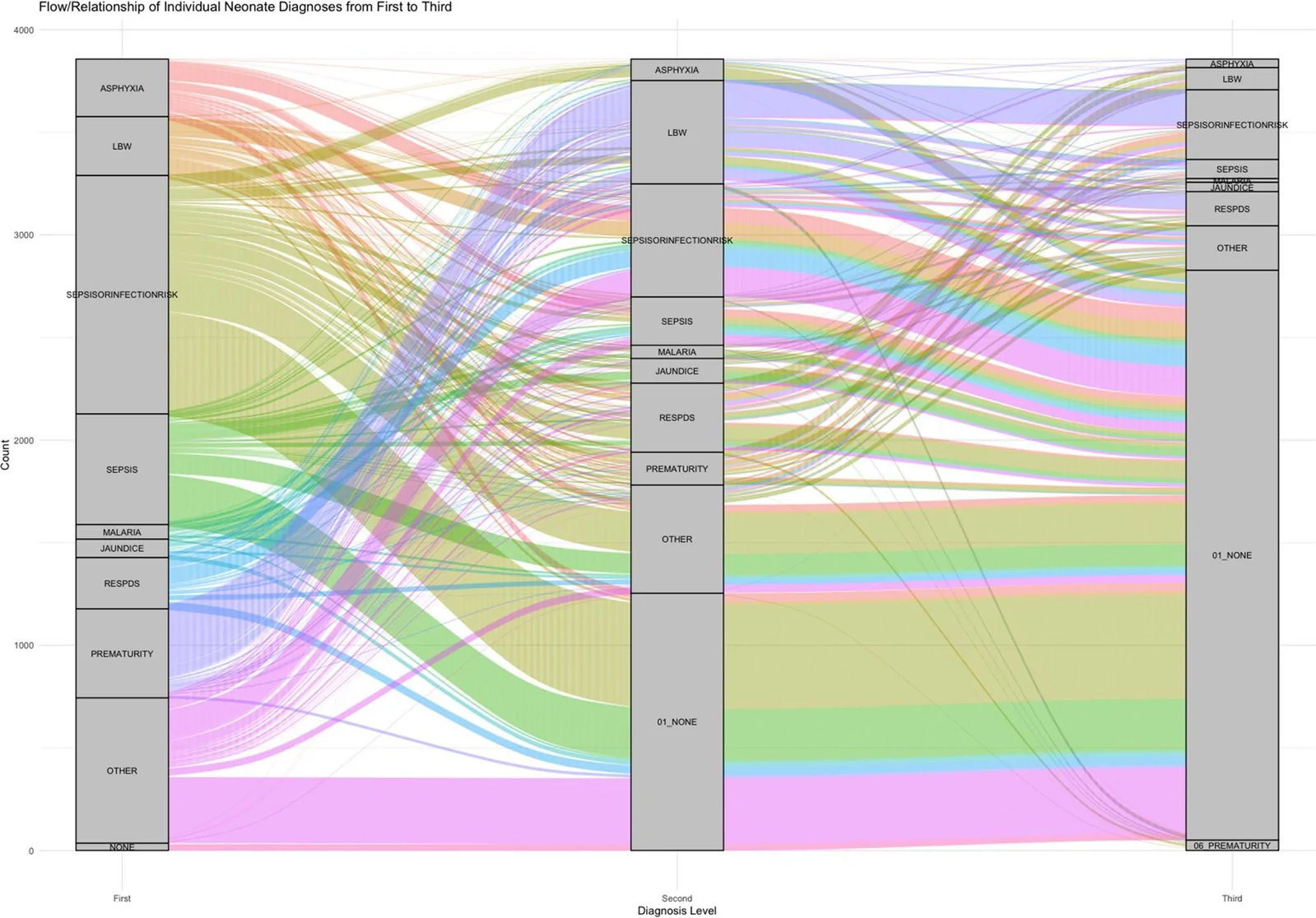
Relationship between primary and other diagnoses among neonates with a primary diagnosis significantly associated with survival status

## Data Availability

Data for this manuscript was made available to us by the Kono District Special Care Baby Unit. Data are not publicly available though may be requested from the Sierra Leone Ethics and Scientific Review Committee. It would breach compliance with the protocol approved by our research ethics board to make the data publicly available. Contact the corresponding author for additional information.

## Appendix

**Table 5:** Summary of neonate diagnoses

Diagnosis as Analyzed	Diagnosis as Recorded *
Birth Asphyxia	Birth Asphyxia I
	Birth Asphyxia II
	Birth Asphyxia III
Low Birthweight	Low Birthweight
Very Low Birthweight	Very Low Birthweight
Extremely Low Birthweight	Extremely Low Birthweight
HIV Exposure	HIV Exposure
Risk of Neonatal Sepsis or Infection	Risk of Neonatal Sepsis
	Risk of Neonatal Infection
Neonatal Sepsis	Neonatal Sepsis
Late Neonatal Sepsis	Late Neonatal Sepsis
Malaria	Malaria
Neonatal Jaundice	Neonatal Jaundice
Meningitis	Meningitis
Macrosomia	Macrosomia
Respiratory Distress Syndrome	Respiratory Distress Syndrome
Prematurity	Prematurity
Other^**^	Other^**^
	Hyperthermia

*Highly related variables were combined into one level on their suggestions based on clinical practice at the site

**“Other” is a dx recorded by SCBU providers in patient charts and includes congenital abnormalities, birth injuries, acute respiratory infection, and other less common diagnoses

**Table 6:** Neonate survival vs. death by primary diagnosis upon SCBU admission

	Survival *N*=3,298 (94.63%)	Death *N*=187(5.37%)	OR	95% CI	*p*-value*
Birth Asphyxia					<0.001
Yes	201 (80.04%)	44 (17.96%)	0.211	0.147, 0.307	
No	3,097 (95.62%)	142 (4.38%)	*ref*	*ref*	
Low Birthweight					0.875
Yes	203 (94.42%)	12 (5.58%)	0.957	0.546, 1.840	
No	3,095 (94.65%)	175 (5.35%)	*ref*	*ref*	
Very Low Birthweight					0.005
Yes	34 (82.93%)	7 (17.07%)	0.268	0.124, 0.666	
No	3,264 (94.77%)	180 (5.23%)	*ref*	*ref*	
Extremely Low Birthweight					1
Yes	4 (100.00%)	0 (0.00%)	*NA*	*NA*	
No	3,294 (94.63%)	187 (5.37%)	*NA*	*NA*	
HIV Exposure					0.369
Yes	56 (98.25%)	1 (1.75%)	3.213	0.702, 56.966	
No	3,242 (94.57%)	186 (5.43%)	*ref*	*ref*	
Risk of Neonatal Sepsis or Infection					<0.001
Yes	1,026 (97.62%)	25 (2.38%)	2.926	1.945, 4.592	
No	2,272 (93.31%)	163 (6.69%)	*ref*	*ref*	
Neonatal Sepsis					0.088
Yes	488 (96.25%)	19 (3.75%)	1.536	0.971, 2.571	
No	2,810 (94.36%)	168 (5.64%)	*ref*	*ref*	
Malaria					0.585
Yes	67 (97.10%)	2 (2.90%)	1.918	0.596, 11.738	
No	3,231 (94.58%)	185 (5.42%)	*ref*	*ref*	
Neonatal Jaundice					0.626
Yes	82 (96.47%)	3 (3.53%)	1.564	0.578, 6.416	
No	3,216 (94.59%)	184 (5.41%)	*ref*	*ref*	
Meningitis					1
Yes	9 (100.00%)	0 (0.00%)	*NA*	*NA*	
No	3,289 (94.62%)	187 (5.38%)	*NA*	*NA*	
Macrosomia					1
Yes	28 (96.55%)	1 (3.45%)	1.593	0.337, 28.474	
No	3,270 (94.61%)	186 (5.82%)	*ref*	*ref*	
Respiratory Distress Syndrome					0.227
Yes	225 (96.57%)	8 (3.43%)	1.638	0.850, 3.665	
No	3,073 (94.50%)	179 (5.50%)	*ref*	*ref*	
Prematurity					<0.001
Yes	318 (90.08%)	35 (9.92%)	0.463	0.319, 0.691	
No	2,980 (95.15%)	152 (4.85%)	*ref*	*ref*	
Other					0.759
Yes	528 (94.96%)	28 (5.04%)	1.082	0.728, 1.667	
No	2,770 (94.57%)	159 (5.43%)	*ref*	*ref*	

**p*-value bolded if <0.05

## References

[1] World Bank Group. Mortality rate, neonatal (per 1,000 live births), Sub-Saharan Africa. World Bank Open Data. 2021. https://data.worldbank.org. Accessed August 17, 2023.

[2] Neonatal mortality. UNICEF DATA. 2025. https://data.unicef.org/topic/child-survival/neonatal-mortality/. Accessed July 28.

[3] Hug L, Alexander M, You D, Alkema L. National, regional, and global levels and trends in neonatal mortality between 1990 and 2017, with scenario-based projections to 2030: a systematic analysis. Lancet Glob Health. 2019;7(6):e710–20. 10.1016/S2214-109X(19)30163-9. PMC6527519 31097275

[4] World Health Organization. Child mortality: Neonatal mortality rate. World Health Organization: Data. May 16. 2024. https://data.who.int/indicators/i/E3CAF2B/A4C49D3. Accessed July 28, 2025.

[5] Rosa-Mangeret F, Benski AC, Golaz A, et al. 2.5 Million Annual Deaths—Are Neonates in Low- and Middle-Income Countries Too Small to Be Seen? A Bottom-Up Overview on Neonatal Morbi-Mortality. Trop Med Infect Dis. 2022;7(5):5. 10.3390/tropicalmed7050064. PMC9148074 35622691

[6] Sharrow D, Hug L, You D, et al. Global, regional, and national trends in under-5 mortality between 1990 and 2019 with scenario-based projections until 2030: a systematic analysis by the UN Inter-agency Group for Child Mortality Estimation. Lancet Glob Health. 2022;10(2):e195–206. 10.1016/S2214-109X(21)00515-5. PMC8789561 35063111

[7] StatsSL SSL. ICF. Sierra Leone Demographic and Health Survey 2019. Published online October 1, 2020. https://dhsprogram.com/publications/publication-FR365-DHS-Final-Reports.cfm. Accessed July 28, 2025.

[8] Sserwanja Q, Nuwabaine L, Kamara K, Musaba MW. Prevalence and factors associated with utilisation of postnatal care in Sierra Leone: a 2019 national survey. BMC Public Health. 2022;22(1):102. 10.1186/s12889-022-12494-5. PMC8760783 35031038

[9] Liu L, Oza S, Hogan D, et al. Global, regional, and national causes of under-5 mortality in 2000–15: an updated systematic analysis with implications for the Sustainable Development Goals. Lancet. 2016;388(10063):3027–35. 10.1016/S0140-6736(16)31593-8. PMC5161777 27839855

[10] Fottrell E, Osrin D, Alcock G, et al. Cause-specific neonatal mortality: analysis of 3772 neonatal deaths in Nepal, Bangladesh, Malawi and India. Arch Dis Child - Fetal Neonatal Ed. 2015;100(5):F439–47. 10.1136/archdischild-2014-307636. PMC4552925 25972443

[11] Shefali Oza JE, Lawn DR, Hogan C, Mathers, Simon N, Cousens. Neonatal cause-of-death estimates for the early and late neonatal periods for 194 countries: 2000–2013. Bull World Health Organ. 2015;9319–28. 10.2471/BLT.14.139790. PMC4271684 25558104

[12] Carshon-Marsh R, Aimone A, Ansumana R, et al. Child, maternal, and adult mortality in Sierra Leone: nationally representative mortality survey 2018–20. Lancet Glob Health. 2021;10(1):e114–23. 10.1016/S2214-109X(21)00459-9. PMC8672062 34838202

[13] Sesay U, Sheriff SD, Osborne A. Community health workers role in combating public health emergencies: lessons from Sierra Leone. Discov Health Syst. 2025;4(1):1–7. 10.1007/s44250-025-00210-0.

[14] Pieterse P, Saracini F. Unsalaried health workers in Sierra Leone: a scoping review of the literature to establish their impact on healthcare delivery. Int J Equity Health. 2023;22:255. 10.1186/s12939-023-02066-3. PMC10709924 38066622

[15] Pieterse P, Saracini F. Employed but Unpaid, Volunteers or Paradoxical Surplus? Sierra Leone’s Unsalaried Health Workforce. Int J Health Plann Manage. 2025. 10.1002/hpm.70016. PMC12794118 40778790

[16] Koroma MM, Kamara MA, Keita N, Lokossou VK, Sundufu AJ, Jacobsen KH. Access to Essential Medications and Equipment for Obstetric and Neonatal Primary Care in Bombali District, Sierra Leone. World Med Health Policy. 2019;11(1):8–23. 10.1002/wmh3.295.

[17] Oyerinde K, Amara P, Harding Y. Barriers to Uptake of Emergency Obstetric and Newborn Care Services in Sierra Leone: A Qualitative Study. J Community Med Health Educ Published online September. 2022;16. 10.4172/2161-0711.1000149.

[18] Osborne A, Bai-Sesay AU, Bangura C, Rogers H, Ahinkorah BO. Socio-economic and geographical inequalities in neonatal mortality rates in Sierra Leone, 2008–2019. BMC Pediatr. 2024;24(1):1. 10.1186/s12887-024-05189-w. PMC11585221 39578763

[19] Sserwanja Q, Mutisya LM, Nuwabaine L, Kamara K, Mutebi RK, Musaba MW. Continuum of maternal and newborn health in Sierra Leone: a 2019 national survey. Arch Public Health. 2022;80:186. 10.1186/s13690-022-00946-8. PMC9364565 35945622

[20] Turan J, Ojengbede O, Fathalla M, et al. Positive Effects of the Non-pneumatic Anti-shock Garment on Delays in Accessing Care for Postpartum and Postabortion Hemorrhage in Egypt and Nigeria. J Womens Health. 2011;20(1):91–8. 10.1089/jwh.2010.2081. PMC3052289 21190486

[21] Proos R, Mathéron H, Vas Nunes J, et al. Perspectives of health workers on the referral of women with obstetric complications: a qualitative study in rural Sierra Leone. BMJ Open. 2020;10(12):e041746. 10.1136/bmjopen-2020-041746. PMC7733167 33303460

[22] Saffa G, Keimbe C, Bangalie A, et al. Factors contributing to delays in accessing health facility-based maternal delivery services in Sierra Leone, 2018: A community-based cluster survey. PLoS ONE. 2024;19(9):e0307179. 10.1371/journal.pone.0307179. PMC11407616 39288131

[23] Caviglia M, Putoto G, Conti A, et al. Association between ambulance prehospital time and maternal and perinatal outcomes in Sierra Leone: a countrywide study. BMJ Glob Health. 2021;6(11). 10.1136/bmjgh-2021-007315. PMC8634006 34844999

[24] Maternal N, Child and Adolescent Health | UNICEF Sierra Leone. 2025. https://www.unicef.org/sierraleone/maternal-neonatal-child-and-adolescent-health. Accessed July 28,

[25] No Small Thing: Improved Care for Newborns in Sierra Leone | Partners In Health. 2025. https://www.pih.org/article/no-small-thing-improved-care-newborns-sierra-leone. Accessed July 28.

[26] Making strides to improve maternal health in Sierra Leone | UNICEF. 2025. https://www.unicef.org/stories/making-strides-maternal-health-worst-place-to-be-mother. Accessed July 28.

[27] Luke RD, Akhigbe IE, Bell NV, Sovula HS, Koroma MJ, Jonjo DA. Survival status and predictors of mortality among preterm neonates admitted to a tertiary hospital in Sierra Leone. J Afr Neonatol. 2024;2(4):89–96.

[28] Conroy N, Barr DA, Nalley J, Conteh JEM, Mitchell L, Bury G. Electrification and specialist training associated with decreased neonatal mortality and increased admissions in Sierra Leone. Acta Paediatr. 2025;114(2):340–5. 10.1111/apa.17431. PMC11706763 39305007

[29] RStudio Team. RStudio: Integrated Development Environment for R. Published online. 2022. http://www.rstudio.com/.

[30] Healthy Newborn Network. Healthy Newborn Network. 2026. https://healthynewbornnetwork.org/country/benin/. Accessed January 30.

[31] Taye K, Kebede Y, Tsegaw D, Ketema W. Predictors of neonatal mortality among neonates admitted to the neonatal intensive care unit at Hawassa University Comprehensive Specialized Hospital, Sidama regional state, Ethiopia. BMC Pediatr. 2024;24(1):237. 10.1186/s12887-024-04689-z. PMC10988874 38570750

[32] Sackey AH, Tagoe LG. Admissions and mortality over a 5-year period in a limited-resource neonatal unit in Ghana. Ghana Med J. 2019;53(2):117–25. 10.4314/gmj.v53i2.6. PMC6697770 31481807

[33] Lakoh S, Bangura MM, Adekanmbi O, et al. Impact of COVID-19 on the Utilization of HIV Testing and Linkage Services in Sierra Leone: Experience from Three Public Health Facilities in Freetown. AIDS Behav. 2024;28(4):1235–43. 10.1007/s10461-023-04149-2. PMC10940454 37642824

[34] Vandy A, Lahai M, Bangura AR, et al. Admission pattern and outcome of hypertensive disorders of pregnancy: a retrospective study of admitted cases at the Princess Christian Maternity Hospital in Freetown, Sierra Leone. BMC Pregnancy Childbirth. 2025;25(1):890. 10.1186/s12884-025-08059-0. PMC12382171 40859269

[35] Stitterich N, Shepherd J, Koroma MM, Theuring S. Risk factors for preeclampsia and eclampsia at a main referral maternity hospital in Freetown, Sierra Leone: a case-control study. BMC Pregnancy Childbirth. 2021;21(1):413. 10.1186/s12884-021-03874-7. PMC8173903 34078312

[36] Annan GN, Asiedu Y. Predictors of Neonatal Deaths in Ashanti Region of Ghana: A Cross-Sectional Study. Adv Public Health. 2018;2018(1):9020914. 10.1155/2018/9020914.

[37] Pain D, MacDuffie E, Martei YM, et al. Barriers to Implementing a Quality Improvement Program in Low- and Middle-Income Countries: Adequacy of Resources. JCO Glob Oncol. 2024;10e2400114. 10.1200/GO.24.00114. PMC11529835 39447091

